# Antimicrobial Resistance and Clonal Lineages of *Staphylococcus aureus* from Cattle, Their Handlers, and Their Surroundings: A Cross-Sectional Study from the One Health Perspective

**DOI:** 10.3390/microorganisms10050941

**Published:** 2022-04-30

**Authors:** Vanessa Silva, Susana Correia, Jaqueline Rocha, Célia M. Manaia, Adriana Silva, Juan García-Díez, José Eduardo Pereira, Teresa Semedo-Lemsaddek, Gilberto Igrejas, Patrícia Poeta

**Affiliations:** 1 Microbiology and Antibiotic Resistance Team (MicroART), Department of Veterinary Sciences, University of Trás-os-Montes and Alto Douro (UTAD), 5000-801 Vila Real, Portugal; vanessasilva@utad.pt (V.S.); scorreia@utad.pt (S.C.); adrianaa.silva95@gmail.com (A.S.); 2 Department of Genetics and Biotechnology, University of Trás-os-Montes and Alto Douro (UTAD), 5000-801 Vila Real, Portugal; gigrejas@utad.pt; 3 Functional Genomics and Proteomics Unit, University of Trás-os-Montes and Alto Douro (UTAD), 5000-801 Vila Real, Portugal; 4 Associated Laboratory for Green Chemistry (LAQV-REQUIMTE), University NOVA of Lisboa, 1099-085 Lisbon, Portugal; 5 Centro de Biotecnologia e Química Fina (CBQF), Laboratório Associado, Escola Superior de Biotecnologia, Universidade Católica Portuguesa, 4169-005 Porto, Portugal; jrocha@ucp.pt (J.R.); cmanaia@ucp.pt (C.M.M.); 6 Veterinary and Animal Research Centre (CECAV), University of Trás-os-Montes and Alto Douro (UTAD), 5000-801 Vila Real, Portugal; juangarciadiez@gmail.com (J.G.-D.); jeduardo@utad.pt (J.E.P.); 7 Associate Laboratory for Animal and Veterinary Sciences (AL4AnimalS), University of Trás-os-Montes and Alto Douro (UTAD), 5000-801 Vila Real, Portugal; 8 Centro de Investigação Interdisciplinar em Sanidade Animal (CIISA), Faculdade de Medicina Veterinária, Avenida da Universidade Técnica, Universidade de Lisboa, 1300-477 Lisboa, Portugal

**Keywords:** *Staphylococcus aureus*, cattle, cows, transmission

## Abstract

*Staphylococcus aureus* have been progressively identified in farm animals and in humans with direct contact with these animals showing that *S. aureus* may be a major zoonotic pathogen. Therefore, we aimed to isolate *S. aureus* from cows, their handlers, and their immediate surroundings, and to investigate the antimicrobial resistance and genetic lineages of the isolates. Mouth and nose swabs of 244 healthy cows (195 Maronesa, 11 Holstein-Friesians, and 28 crossbreeds), 82 farm workers, 53 water and 63 soil samples were collected. Identification of species was carried out by MALDI-TOF MS Biotyper. The presence of antimicrobial resistance genes and virulence factors was assessed based on gene search by PCR. All isolates were typed by multilocus sequence typing and *spa*-typing. From 442 samples, 33 (13.9%), 24 (29.3%), 1 (2%), and 1 (2%) *S. aureus* were recovered from cows, farm workers, water, and soil samples, respectively. Most of the isolates showed resistance only to penicillin. *S. aureus* isolates were ascribed to 17 sequence types (STs) and 26 *spa*-types. Some clonal lineages were common to both cows and farm workers such as ST30-t9413, ST72-t148, and ST45-t350. Through a One Health approach, this study revealed that there is a great diversity of clonal lineages of *S. aureus* in cows and their handlers. Furthermore, some *S. aureus* lineages are common to cows and handlers, which may suggest a possible transmission.

## 1. Introduction

Zoonotic disease events have highlighted the increasing effect of pathogens on human and animal health [1,2]. Therefore, in the past, the One Medicine concept was implemented, which aimed to address animal–human interactions and human and animal health [3]. Later, however, it was evident that the environment was also directly related to human and animal health through, for example, agricultural intensification, climate change, human encroachment into wildlife habitats, and environmental contamination, which were recognized as drivers for zoonotic disease emergence threatening human and animal populations [2,4]. Therefore, a collaborative and multi-disciplinary approach, involving human–animal–environment interactions has been implemented in order to understand the ecology of emerging zoonotic diseases [5]. The One Health concept focuses on the relatedness of human, animal, and environmental health focusing on the emerging zoonoses, food safety, and antimicrobial resistance [5,6,7]. Antimicrobial resistance has been included by the World Health Organization in top ten threats to global health in 2019 and has been recognized as a One Health issue since it can arise in humans, animals, and the environment, and can spread from one compartment to another, between regions and countries [5,8]. A One Health approach to antimicrobial resistance aims not only at understanding this issue, but also how it spreads across hospitals, communities, farming animals, pets, wild animals, wastewaters, and natural water reservoirs [9].

*Staphylococcus aureus* are part of the skin and mucous membranes of humans and animals, with humans being the main reservoir [10]. However, *S. aureus* also comprises opportunist bacteria that cause multiple infections, including skin and soft tissue infections, bacteremia, osteomyelitis, endocarditis, among others [11]. *S. aureus,* particularly methicillin-resistant *S. aureus* (MRSA), infections have become increasingly difficult to treat due to their ability to easily acquire antimicrobial resistance determinants [12]. In fact, *S. aureus* is resistant to almost all antimicrobials available so far [13]. Furthermore, *S. aureus* produces an enormous variety of virulence factors which include a wide range of toxins and immune evasion factors [14]. *S. aureus* is a widespread species that has been isolated from humans, hospital settings, farm animals, pets, wild animals, wastewater, and surface water [12,15,16,17,18,19,20]. *S. aureus* isolates can be grouped into different genetic lineages defined by molecular typing methods, such as multilocus sequence typing (MLST), *spa*-typing, and whole genome sequencing [21]. Epidemiological studies have suggested that these lineages are well adapted to their respective host [22,23]. For instance, several *S. aureus* clonal complexes (CCs), which are defined by MLST, have been detected in only one animal group as is the case of CC522 and CC385, which have been found only in small ruminants and avian species, respectively [23,24,25]. However, host shifts are a natural feature of *S. aureus* evolution. *S. aureus* CCs found in different species may reflect intraspecies transmission or a broad host range [23]. *S. aureus* isolated from healthy and infected human are mainly represented by CC1, CC5, CC8, CC12, CC15, CC22, CC25, CC30, CC45, CC51, and CC121 [26]. Regarding *S. aureus* from animals, CC1, CC5, CC9, CC45, CC97, and CC398 are the most frequently detected [22]. However, it is important to point out that some dominant MRSA lineages differ from dominant MSSA lineages in each host [22]. Healthy bovine are carriers of *S. aureus* mainly in the teat skin, nasal cavity, and rectum [27]. *S. aureus,* particularly *S. aureus* CC97, is also a frequent etiological agents of mastitis in cows [27]. Close contact between bovine and farm workers may promote transmission of strains in both ways [28,29]. In fact, studies have shown that CC97 subclades for human infection originated in bovine-to-human host dissemination, which indicates that animals may act as *S. aureus* reservoirs that can spread to humans [27,30]. The autochthonous Maronesa cattle is a traditional Portuguese breed used for meat production commercialized with PDO—Protected Designation of Origin [31,32]. Maronesa cattle is considered a threatened breed that has been used for centuries in agricultural work [32]. Therefore, in this study, we isolated *S. aureus* from farm workers, cows, and their environments and aimed to find evidence of bacterial transmission and spread investigating the antimicrobial resistance and genetic lineages of the isolates.

## 2. Materials and Methods

### 2.1. Sample Collection

A total of 442 samples were collected from 64 farms in the North of Portugal, which comprises 244 cows (195 Maronesa breed, 11 Holstein-Friesian and 28 crossbreed), 82 farm workers, 53 water samples, and 63 soil samples from February to April 2019. Samples from cows and farm workers were collected with a nasal and mouth swab (one sample per individual). The farms are managed by families who are dedicated to agriculture and generally share the same household. Water samples were collected from the cows’ drinkers using sterile 500 mL plastic bottles with sodium thiosulfate and preserved at 4–8 °C. All samples were filtered on the same day they were collected. Soil samples were collected from the farm grounds with a sterile plastic bag. The age of the cows ranged from 4 to 22 years with an average of 10 years and among the 244 cows, 229 were females, and 15 males (Supplementary Table S1).

### 2.2. S. aureus Isolation

The swabs and 2 g of soil sample were inserted into tubes containing 5 mL of Brain Heart Infusion (BHI) broth (LiofilChem, Via Scozia, Italy) with 6.5% of NaCl and incubated at 36 °C for 24 h. Then, the inoculum was seeded onto Baird–Parker agar (Oxoid, Basingstoke, UK) plates for *S. aureus* isolation. Water samples were filtered through a cellulose nitrate 0.45 μm pore membrane filter (Whatman, Maidstone, UK). The filters were then inserted into tubes BHI broth tubes 6.5% of NaCl and incubated at 37 °C for 24 h. After the incubation period, the inoculum was seeded onto Baird–Parker agar plates. Colonies, with *S. aureus* characteristics but showing morphological differences, were collected from each plate. *S. aureus* species identification was performed by biochemical tests (catalase, DNase and coagulase) and by MALDI-TOF MS Biotyper (Bruker Daltonics, Billerica, MA, USA).

### 2.3. Antimicrobial Susceptibility Testing

Antibiotic susceptibility was carried out in all *S. aureus* isolates and their susceptibility profile was determined using a Kirby–Bauer disk diffusion method against the following 14 antimicrobial agents (concentration/disk; Oxoid, Basingstoke, UK)): penicillin (1U), cefoxitin (30 μg), chloramphenicol (30 μg), ciprofloxacin (5 μg), clindamycin (2 μg), erythromycin (15 μg), fusidic acid (10 μg), gentamicin (10 μg), kanamycin (30 μg), linezolid (10 μg), mupirocin (200 μg), tetracycline (30 μg), tobramycin (10 μg), and trimethoprim/sulfamethoxazole (1.25/23.75 μg). The determination and interpretation of the results was made according to the European Committee on Antimicrobial Susceptibility Testing (EUCAST, 2018) standards except for kanamycin that followed the Clinical and Laboratory Standards Institute guidelines (CLSI, 2017). *S. aureus* strain ATCC 25923 was used as quality control in all assays. 

### 2.4. Antimicrobial Resistance and Virulence Genes

DNA extraction was performed as previously described using lysostaphin and proteinase K (Sigma Aldrich, St. Louis, MI, USA) [33]. All isolates were screened for the presence antimicrobial resistance genes by PCR and sequencing according to their phenotypic resistance: penicillin (*bla*Z), aminoglycosides (*aac*(6′)-Ie-*aph*(2″)-Ia, *aph*(3′)-IIIa, *ant*(4′)-Ia and *str*), macrolides and lincosamides (*erm*A, *erm*B, *erm*C, *erm*T, *mph*C, *msr*(A/B), *lnu*A, *lnu*B, *vga*A and *vga*B), fusidic acid (*fus*B, *fus*C and *fus*D), tetracyclines (*tet*M, *tet*L, *tet*K and *tet*O) and chloramphenicol (*fex*A, *fex*B, *cat_pC194_*, *cat*_pC221_ and *cat*_pC223_) (Table S2). The presence of the virulence genes *luk*F/*luk*S-PV (Panton–Valentine Leukocidin), *hla*, *hlb* and *hld* (alpha-, beta- and delta-hemolysins), *eta* and *etb* (exfoliative toxins), and *tst* (toxic shock syndrome toxin) was also investigated by PCR. In addition, all isolates were screened for the presence of the *scn* gene, which is a marker of the immune evasion cluster (IEC) system. In isolates positive for *scn*, the presence of the *chp*, *sak*, *sea* and *sep* genes was assessed to determine the IEC group [34]. Positive and negative controls used in all experiments belonged to the strain collection of the University of Trás-os-Montes and Alto Douro.

### 2.5. Molecular Typing

All isolates were typed by MLST, *spa*-, and *agr*-typing. The *spa* region was amplified by PCR, the fragments sequenced, and the obtained sequences were analyzed using Ridom^®^ Staph-type software (version 1.5, Ridom GmbH, Würzburg, Germany) [35]. MLST genotyping was performed as previously described [36]. Allele and STs were determined using the *Staphylococcus* MLST database at https://pubmlst.org/ (accessed on 7 October 2021). Isolates were also characterized by *agr*-typing (I–IV) by PCR using specific primers and conditions [37].

## 3. Results

In this study, *S. aureus* strains were isolated from cows, farm workers, and the cows’ surrounding environment (soil and water). *S. aureus* were found in 24 (37.5%) of the 64 farms included in this study. A total of 58 (13.1%) *S. aureus* were isolated from the 442 samples. From the 244 cows sampled, 32 (13.1%) were colonized by *S. aureus*. However, one cow co-carried two different lineages of *S. aureus*; thus, 33 *S. aureus* were isolated from cows. Among the three tested breeds, Maronesa, Holstein-Friesian and crossbreed, *S. aureus* were detected in 25. 2 and 6, respectively. Regarding the farm workers, 24 (29.3%) *S. aureus* were recovered from the 82 samples. Water and soil samples were collected from 53 cows’ drinkers and 63 soil grounds and only one isolate of each origin was recovered.

**Figure 1:**
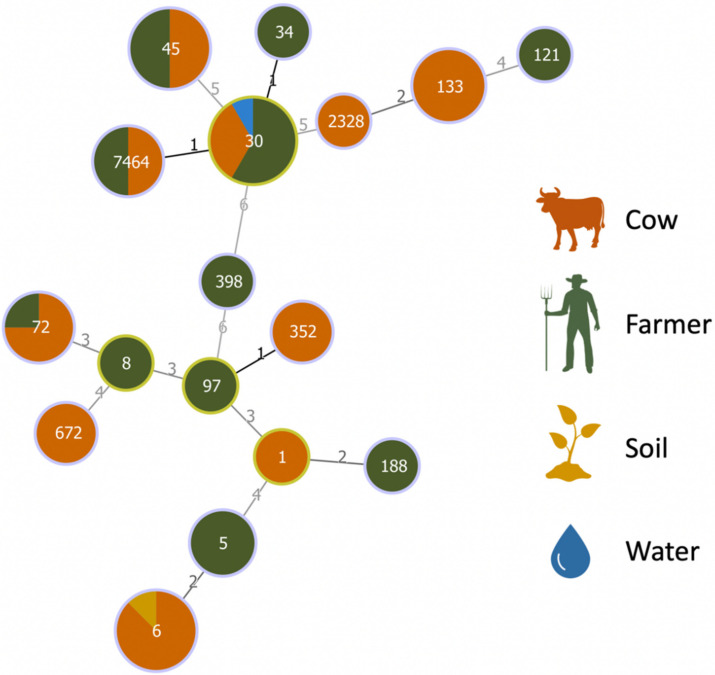
Minimum spanning tree, based on MLST of 58 *S. aureus* isolated from farm workers, cows and their surroundings. The minimum spanning tree graph (MST) was created with PHYLOViZ using the goeBURST algorithm. The dominant STs are represented by the circles with larger diameters. Each color represents one sample source. Numbers on lines indicate locus variants between adjacent nodes.

Table 1 shows the percentage of *S. aureus* isolates resistant and susceptible to each antibiotic. Farms with positive samples are listed in Table 2 as well as the resistance and virulence profiles, and clonal lineages of the isolates. In 8 (Farm 3, 10, 15, 17, 39, 42, 60 and 63) of the 24 positive farms, *S. aureus* was isolated from cows only and, in seven farms (Farm 6, 20, 25, 46, 48, 56, and 58), it was isolated from farm workers only. Interestingly, in farms 14 and 55, *S. aureus* was isolated only from soil and water samples, respectively. In the remaining farms, *S. aureus* was isolated from both cows and farm workers. All isolates were characterized regarding their antimicrobial resistance, virulence, and clonal lineages. Nineteen isolates from cows (*n* = 15), farm workers (*n* = 2), soil (*n* = 1), and water (*n* = 1) were susceptible to all antibiotics tested (Table 2). Multidrug resistance was found in one isolate from a cow (VS3222) and one from a human (VS3263). Resistance to penicillin was detected in 36 isolates and all carried the *bla*Z gene. Four isolates were resistant to aminoglycosides and harbored the *aac*(6′)*-aph*(2″) (*n* = 4), *aph*(3′)-IIIa (*n* = 3) and *str* genes. Two isolates from cows and one from a worker showed resistance to tetracycline conferred by the *tet*K gene. Resistance to erythromycin was found in four *S. aureus* isolated from farm workers with two being co-resistant to clindamycin. Resistance to macrolides and lincosamides was encoded by the *erm*C (*n* = 2), *erm*T, and *erm*B. Only one isolate showed phenotypic resistance to fusidic acid, but none of the tested genes were present. Finally, two *S. aureus* isolated from a cow and its handler carried the *cat_pC221_* gene, which is responsible for chloramphenicol resistance. Five isolates from cows and one from one farm worker were positive for the *scn* gene of the IEC system and were further investigated regarding the presence of the other IEC genes. The isolates were ascribed to IEC type B (*n* = 4), G (*n* = 2) and E. All isolates harbored the virulence genes *hla* and *hld*. As expected, the *hlb* gene was detected in all IEC-negative isolates (*n* = 56), and six isolates also carried the *tst* gene. All isolates were typed by MLST, *spa*- and *agr*-type. The 58 isolates were affiliated to 18 STs and 26 different *spa*-types, with 6 and 12 distinct STs and *spa*-types for the bovine isolates and nine distinct STs and *spa*-types for human isolates (Figure 1). The most common *S. aureus* lineage in cows was ST6- t18899 (*n* = 9/244) and in farm workers was ST30-t012 (*n* = 7/82). In general, *S. aureus* isolates from cows were ascribed to ST6 (*n* = 9), ST133 (*n* = 5), ST30 (*n* = 4), ST45 (*n* = 4), ST72 (*n* = 3), ST672 (*n* = 2), ST7464 (*n* = 2), ST352 (*n* = 2), ST1 and ST2328, and *spa*-types t16615 (*n* = 9), t9413 (*n* = 4), t18899 (*n* =3), t148 (*n* = 3), t959 (*n* = 2), t871 (*n* = 2), t3750, t2207, t7355, t7669, t4735, t350, t706, t015, t267, t359 and t563. Isolated from farm workers belonged to ST30 (*n* = 7), ST45 (*n* = 4), ST5 (*n* = 3), ST72, ST121, ST97, ST34, ST188, ST8 and ST398, and *spa*-types t012 (*n* = 5), t9413 (*n* = 2), t018 (*n* = 2), t045 (*n* = 2), t189 (*n* = 2), t148, t7669, t350, t015, t018, t002, t162, t414, t008 and t571. The two *S. aureus* isolates from water and soil were ascribed to ST30-t018 and ST6-t16615, respectively. As for *agr*-typing, the isolates were grouped into *agr* type I (*n* = 35), II (*n* = 3), III (*n* = 19), and IV (*n* = 1).

Evidence of a possible transmission of *S. aureus* between farm workers and cows is shown in Farms 16, 62, and 64. For instance, in Farm 16, the same clonal lineage ST30-t9413 in isolates from four cows and one worker, and all isolates have the same phenotype and genotype. In addition, isolates from two farm workers share the same linages which may also suggest a possible human-to-human transmission since workers from the same farm are related and share the same household, and the same is observed in Farm 13. Transmission between cows sharing the same environment may also occur. In Farms 10, 13, and 60, *S. aureus* from cows share the same clonal lineages among them, which possibly indicates a cow-to-cow transmission.

## 4. Discussion

Transmission of *S. aureus* between cows and people working with dairy cattle has been reported in 2007 [38]. Since then, many studies have been published with dairy cattle and the possible transmission between cows and farm workers [29,38,39,40]. However, the great majority of studies focus only on *S. aureus* as a cause of bovine mastitis or its presence in bovine milk. In fact, *S. aureus* causing mastitis and the transmission to and from farm workers through direct contact have been extensively studied [29,40]. Indeed, studies investigating the presence of *S. aureus* in healthy beef cattle and the animal–human–environment transmission in the One Health context are scarce. In our study, we collected a total of 442 samples from cows, farm workers, and the farm environment (soil and water). In our previous study, we reported the absence of MRSA in Maronesa cattle, and so this is the first study reporting the presence of *S. aureus* in Maronesa cattle, which is an important traditional Portuguese breed [32]. From the 244 cows sampled, 13.1% were colonized by *S. aureus*, which is higher than most studies conducted with healthy cattle. Other studies conducted with healthy cattle reported an *S. aureus* frequency of between 5% and 8% [41,42,43]. Moreover, a study carried out in Tunisia reported an even lower frequency of *S. aureus* of only 1.3% in healthy cattle [44]. Likewise, Garipcin et al. investigated the presence of *S. aureus* in healthy cattle and humans in close contact with these animals and found a prevalence of 3.2% and 29.3% in cattle and humans, respectively [45]. The results of this study, in relation to samples of human handlers, is the same as that obtained in our study (29.3%). In fact, it has been reported that *S. aureus* is part of the normal mucosa of around 30% of the human population [46]. In contrast, another study carried out with samples from cattle and their caretakers found *S. aureus* in 42.9% and 74.2% in cattle and caretakers, respectively, which is a much higher frequency than most studies including ours [47]. Finally, another study similar to ours, in which the presence of *S. aureus* was investigated in cattle, caretakers, and the farm environment, found *S. aureus* in 4% and 16.6% of animal and human nose samples, but no *S. aureus* was found in the environmental samples. *S. aureus* and MRSA have been reported as environment associated with livestock including pigs, cattle and even in the production chain of dairy products [39,48,49]. In our study, the frequency of *S. aureus* in soil and water was also very low (1.6 and 1.9%, respectively). However, we excepted a higher frequency of *S. aureus* in soil samples since studies have shown that environmental sampling of barns and farms may be used for *S. aureus* and MRSA surveillance in livestock [50,51]. Furthermore, however, there is little information about the survival time of *S. aureus* on soil, and the manure spread on the farm soil could be a source of *S. aureus* on soil surfaces. We also expected to find a higher prevalence of *S. aureus* in the water of the cows’ drinkers since *S. aureus* is present in the mouth and nose of cows and can spread in the water. This low frequency may be due to the *S. aureus* survival rate in fresh water, which was reported to be an average of 2.71 days and 4.84 days at 20 °C and 13 °C, respectively [52].

Zoonotic transmission of *S. aureus* strains between livestock and humans have been reported, particularly, with humans living and working in close contact with a farm [29,39]. *S. aureus* transmission between cattle and farm workers may occur through direct contact or in indirect exposure through the farm environment [39]. In our study, farm environment contamination did not seem to promote *S. aureus* colonization in both cattle and farm workers since only two environmental *S. aureus* were isolated from different farms (farms 14 and 55), and no *S. aureus* was detected in the cows or in the workers of those farms. Potential transmission between cows and workers was detected in farms 16, 49, 53, and 62. In farm 16, all cows were colonized by *S. aureus* ST30-t9413 carrying the *bla*Z, *hla*, *hlb*, and *hld* genes, and one of the farm workers was also colonized by the same *S. aureus* clone harboring the same genes. In addition, two other workers also carried *S. aureus* ST30 but with a different *spa*-type (t018). *S. aureus* ST30 was the predominant clone found in this study and was detected in cows, humans, and soil samples. ST30 is primarily associated with humans but is also spread among livestock, including cows and pigs [53,54]. Furthermore, CC30 comprises the most common MSSA lineage in Europe and gave rise to important epidemic clones such as EMRSA-16 [55,56]. In this study, ST30 isolates were associated with three *spa*-types: t018, t9413, and t012. *S. aureus* from cows were exclusively typed as t9413, while *S. aureus* ST30 from humans were typed as t018, t9413, and t012. *S. aureus* ST30-t012 isolate may be related to the Southwest Pacific clone and was the most prevalent clone among community and hospital settings in Portugal between 1992 and 2011 [56]. ST30-t9413 has only been reported in Portugal in strains isolated from wild owls, superficial waters and one farm worker with close contact with cattle, and all studies were conducted in the same region as this study [19,32,57]. *spa*-type t9413 may be cattle-associated and the ST30-t9413 isolated from farm workers in this study may have an animal origin. Furthermore, CC30 isolates were the only ones carrying the virulence gene *tst*, but none of the ST30-t9413 harbored this gene. The carriage of *tst*, in addition to the hemolysins genes, is in accordance with other studies that have shown that *S. aureus* ST30 often carries pathogenicity islands including *tst* gene [58]. Other *S. aureus* isolated in this study belonged to CC30, such as *S. aureus* ST7464-t871 detected in two cows from farm 39 and *S. aureus* ST34-t414 isolated from a farm worker (farm 47). Another possible piece of evidence of *S. aureus* human-to-animal and animal-to-animal transmission was detected in farms 64 and 60, respectively. All *S. aureus* isolates were typed as ST72 (CC8) and *spa*-type t148. *S. aureus* ST72 was first described in South Korea and is a particularly rare clone elsewhere in the world [59]. However, it is mostly associated with MRSA strains frequently found in the community and hospitals [60]. However, MSSA ST72-t148 has also been reported as a common cause of blood infection in Korea [61]. *S. aureus* ST45 was detected in four cows and four farm workers in this study and associated with five *spa*-types: t015, t7669, t350, t563, and t706. *S. aureus* ST45-t7669 was detected in one cow and one farm worker from farm 62 and, since both isolates encode the same resistance and virulence genes, we can suggest a possible bacterial transmission. *S. aureus* ST45 is a human-associated clone and is a major global MRSA lineage [62]. Nevertheless, MSSA ST45 has been detected in cow mastitis and farm workers with direct contact [63,64]. Effelsberg et al. analyzed a large collection of ST45 isolates from six continents and reported that ST45 phylogeny is defined by two distinct sublineages which correlated with geographical origins of the isolates [62]. However, in our study, since 3 of the 4 ST45 isolates from cows carried the IEC system genes, we can suggest that it may indicate a human spillover rather than an animal-associated ST45 sublineage as previously stated [62]. *S. aureus* ST6-t16615 was the most prevalent lineage in cows and was not detected in human samples. This lineage has been reported among wild rats and owls in Portugal [57,65] and as the main lineage in livestock in Algeria [66]. Although considered a human clone with relatively high prevalence in Asian countries, this lineage seems to be widely disseminated among animals [67]. Furthermore, in our study, none of the isolates carried the IEC systems, which suggests a possible animal adaptation. Some of the remaining *S. aureus* lineages were only detected in cows: ST133, ST672, ST352, ST1, and ST2328. *S. aureus* ST133 and ST2328 belonging to CC133 and ST1 (CC1) are known to be livestock-associated and lately have emerged as important zoonotic lineages [68]. CC133 lineage is regarded as mostly ungulate-animal specific, but it has also been detected in wild animals and surface waters [19,57,69]. In fact, *S. aureus* CC133 has been reported as the most prevalent in bovine mastitis milk [70,71]. *spa*-type t18899, found in three ST133 isolates in our study, was only reported in milk samples [72]. ST672 lineage is an emerging strain from the Indian subcontinent often related with CA-MRSA and rarely found elsewhere [73]. In our study, both ST672 isolates carried the IEC genes and were ascribed to group G, which may confirm a human origin [68]. *S. aureus* ST352 belongs to CC97, which is an animal-specific lineage, but it has also been detected in one farm worker in this study. CC97 is a pandemic bovine *S. aureus* lineage that emerged as a zoonotic agent and has been reported as a human epidemic CA-MRSA after host adaptation [30,47]. Other *S. aureus* lineages were exclusively detected in farm workers such as ST5, ST121, ST188, ST8, and ST398. ST5, ST8, and ST188 classical human linages [47]. However, ST398-t571 is the most common livestock-associated *S. aureus* lineage in Europe [74]. As in animals, the *spa*-type t571 is the most common spa-type in MSSA ST398 in humans [75]. However, this isolate has characteristics typical of being of animal origin: it has resistance to tetracycline conferred by the *tet*K gene, which is known to be a livestock-associated marker, and lacks the IEC system genes, which is currently considered to be the marker for human host adaptation [76]. References [77,78,79,80,81,82,83,84,85,86,87,88,89,90,91] are cited in the Supplementary Materials.

## 5. Conclusions

In this study, both cows and farm workers are carriers of *S. aureus* strains. However, *S. aureus* was isolated from only one soil and one water sample, which may suggest a low survival of *S. aureus* in the environment. Several cow isolates that belonged to classical human genetic lineages were indistinguishable from *S. aureus* isolated from farm workers in close contact with the cows, which suggests a possible transmission from humans as previously evoked. Animal-to-human transmission may have also occurred, although in a smaller number of cases, which indicates an acquisition through occupational contact. Moreover, our results also provide the evidence of *S. aureus* transmission among cows and among humans sharing the same household, although the direction of transfer could not be proven.

## Figures and Tables

**Table 1:** Antimicrobial resistance of 58 positive isolates of *S. aureus*.

Antibiotics	Resistant	Susceptible
	Positive Strains *n* (%)	Positive Strains *n* (%)
Penicillin (1U)	36 (62.1)	22 (37.9)
Chloramphenicol (30 μg)	2 (3.5)	56 (96.5)
Clindamycin (2 μg)	2 (3.5)	56 (96.5)
Erythromycin (15 μg)	4 (6.9)	54 (93.1)
Fusidic acid (10 μg)	1 (1.7)	57 (98.3)
Gentamicin (10 μg)	4 (6.9)	54 (93.1)
Kanamycin (30 μg)	3 (5.2)	55 (94.8)
Tetracycline (30 μg)	3 (5.2)	55 (94.8)
Tobramycin (10 μg)	4 (6.9)	54 (93.1)

**Table 2:** *S. aureus* positive farms, antimicrobial resistance virulence genes, and genetic lineages of the isolates.

Farm	Isolate	Source	Molecular Typing			Antimicrobial Resistance		Virulence Factors	
			ST (CC)	*spa*	*agr*	Phenotype	Genotype	IEC System	Other Genes
3	VS3218	Cow	6 (5)	t16615	I	PEN	*bla*Z		*hla*, *hlb*, *hld*
6	VS3219	Human	45 (45)	t563	I	PEN	*bla*Z		*hla*, *hlb*, *hld*
10	VS3220	Cow	6 (5)	t16615	I	Susceptible			*hla*, *hlb*, *hld*
	VS3221	Cow	6 (5)	t16615	I	FD			*hla*, *hlb*, *hld*
	VS3222	Cow	133 (133)	t4735	I	Susceptible			*hla*, *hlb*, *hld*
13	VS3223	Cow	672	t959	I	PEN, CN, TOB, KAN, TET	*bla*Z, *aac*(6′)-*aph*(2″), *aph*(3′)-IIIa, *tet*K		*hla*, *hlb*, *hld*
	VS3224	Cow	6 (5)	t16615	I	Susceptible			*hla*, *hlb*, *hld*
	VS3225	Cow	6 (5)	t16615	I	PEN, CN, TOB, KAN	*bla*Z, *aac*(6′)-*aph*(2″), *aph*(3′)-IIIa		*hla*, *hlb*, *hld*
	VS3226	Cow	6 (5)	t16615	I	TET	*tet*K		*hla*, *hlb*, *hld*
	VS3227	Human	30 (30)	t012	III	PEN	*bla*Z		*hla*, *hlb*, *hld*, *tst*
	VS3228	Human	30 (30)	t012	III	PEN	*bla*Z		*hla*, *hlb*, *hld*, *tst*
	VS3229	Human	30 (30)	t9413	III	PEN	*bla*Z		*hla*, *hlb*, *hld*
14	VS3230	Soil	6 (5)	t16615	I	Susceptible			*hla*, *hlb*, *hld*
15	VS3231	Cow	6 (5)	t16615	I	Susceptible			*hla*, *hlb*, *hld*
	VS3232	Cow	6 (5)	t16615	I	Susceptible			*hla*, *hlb*, *hld*
16	VS3233	Cow	30 (30)	t9413	III	PEN	*bla*Z		*hla*, *hlb*, *hld*
	VS3234	Cow	30 (30)	t9413	III	PEN	*bla*Z		*hla*, *hlb*, *hld*
	VS3235	Cow	30 (30)	t9413	III	PEN	*bla*Z		*hla*, *hlb*, *hld*
	VS3236	Human	5 (5)	t045	II	PEN	*bla*Z		*hla*, *hlb*, *hld*
	VS3237	Human	97 (97)	t189	I	Susceptible			*hla*, *hlb*, *hld*
	VS3238	Human	30 (30)	t018	III	ERY	*erm*B		*hla*, *hlb*, *hld*, *tst*
	VS3239	Human	30 (30)	t9413	III	PEN	*bla*Z		*hla*, *hlb*, *hld*
	VS3240	Human	30 (30)	t018	III	PEN	*bla*Z		*hla*, *hlb*, *hld*, *tst*
	VS3241	Cow	30 (30)	t9413	III	PEN	*bla*Z		*hla*, *hlb*, *hld*
17	VS3242	Cow	133 (133)	t7355	I	PEN, CN, TOB, KAN	*bla*Z, *aac*(6′)-*aph*(2″), *aph*(3′)-IIIa, *str*		*hla*, *hlb*, *hld*
20	VS3243	Human	5	t002	II	PEN	*bla*Z		*hla*, *hlb*, *hld*
25	VS3244	Human	121 (121)	t162	IV	Susceptible		E	*hla, hld*
39	VS3245	Cow	7464 (30)	t871	III	Susceptible			*hla*, *hlb*, *hld*
	VS3246	Cow	45 (45)	t015	I	PEN		B	*hla, hld*
	VS3247	Cow	352 (97)	t267	I	Susceptible			*hla*, *hlb*, *hld*
	VS3248	Cow	7464 (30)	t871	III	Susceptible			*hla*, *hlb*, *hld*
42	VS3249	Cow	2328 (133)	t3750	III	Susceptible			*hla*, *hlb*, *hld*
46	VS3250	Human	45 (45)	t350	I	PEN	*bla*Z		*hla*, *hlb*, *hld*
47	VS3251	Cow	45 (45)	t706	I	PEN	*bla*Z	B	*hla, hld*
	VS3252	Human	34 (30)	t414	III	PEN	*bla*Z		*hla*, *hlb*, *hld*, *tst*
48	VS3253	Human	45 (45)	t015	I	PEN	*bla*Z		*hla*, *hlb*, *hld*
	VS3254	Human	188 (188)	t189	I	PEN, CN, TOB	*bla*Z, *aac*(6′)-*aph*(2″)		*hla*, *hlb*, *hld*
49	VS3255	Cow	133 (133)	t18899	I	Susceptible			*hla*, *hlb*, *hld*
	VS3256	Cow	133 (133)	t18899	I	Susceptible			*hla*, *hlb*, *hld*
52	VS3257	Cow	672	t959	I	Susceptible		G	*hla, hld*
	VS3258	Cow	672	t959	I	Susceptible		G	*hla, hld*
	VS3259	Human	8	t008	I	PEN, ERY	*bla*Z, *erm*C		*hla*, *hlb*, *hld*
	VS3260	Human	(30)	t012	III	PEN	*bla*Z		*hla*, *hlb*, *hld*
	VS3261	Human	30 (30)	t012	III	PEN	*bla*Z		*hla*, *hlb*, *hld*
55	VS3262	Water	30 (30)	t018	III	Susceptible			*hla*, *hlb*, *hld*, *tst*
56	VS3263	Human	5	45	II	PEN	*bla*Z		*hla*, *hlb*, *hld*
58	VS3264	Human	398	t571	I	PEN, ERY, CD, TET	*bla*Z, *erm*T, tetK		*hla*, *hlb*, *hld*
60	VS3265	Cow	72 (8)	t148	I	PEN	*bla*Z		*hla*, *hlb*, *hld*
	VS3266	Cow	352 (97)	t359	I	PEN	*bla*Z		*hla*, *hlb*, *hld*
	VS3267	Cow	72 (8)	t148	I	PEN	*bla*Z		*hla*, *hlb*, *hld*
62	VS3268	Cow	45 (45)	t350	I	PEN	*bla*Z		*hla*, *hlb*, *hld*
	VS3269	Cow	45 (45)	t7669	I	PEN	*bla*Z	B	*hla, hld*
	VS3270	Human	(30)	t012	III	PEN, ERY, CD	*bla*Z, *erm*C		*hla*, *hlb*, *hld*
	VS3271	Human	45 (45)	t7669	I	PEN	*bla*Z	B	*hla*, *hlb*, *hld*
63	VS3272	Cow	133 (133)	t18899	I	Susceptible			*hla*, *hlb*, *hld*
	VS3273	Cow	1 (1)	t2207	III	Susceptible			*hla*, *hlb*, *hld*
64	VS3274	Cow	72 (8)	t148	I	PEN, C	*bla*Z, *cat_pC221_*		*hla*, *hlb*, *hld*
	VS3275	Human	72 (8)	t148	I	PEN, C	*bla*Z, *cat_pC221_*		*hla*, *hlb*, *hld*

Abbreviations: PEN: Penicillin; CN: gentamycin; TOB: tobramycin; KAN: kanamycin; ERY: erythromycin; CD: clindamycin; TET: tetracycline; C: chloramphenicol; ST: sequence type: CC: clonal complex; IEC: Immune evasion cluster; N.T. not typable.

## Data Availability

Not applicable.

## Supplementary Materials

The following supporting information can be downloaded at: https://www.mdpi.com/article/10.3390/microorganisms10050941/s1, Table S1: Farms, date, and location of sample collection and distribution of the *S. aureus* isolates among cows, farmers, and environment samples; Table S2: Primer pairs used for molecular typing and detection of antimicrobial resistance genes in *S. aureus* strains.
