# Supplementary material for: Antimicrobial Resistance and Clonal Lineages of Staphylococcus aureus from Cattle, Their Handlers, and Their Surroundings: A Cross-Sectional Study from the One Health Perspective
Source: Microorganisms. 2022 Apr 30;10(5):941. doi: 10.3390/microorganisms10050941 (PMC9144820; doi:10.3390/microorganisms10050941)
Supplement: Supplementary file 1 [file microorganisms-10-00941-s001.zip › microorganisms-1678838-supplementary.pdf]

microorganisms

MDPI

Article

Antimicrobial Resistance and Clonal Lineages of *Staphylococcus Aureus* from Cattle, Their Handlers, and Their Surroundings: A Cross-Sectional Study from the One Health Perspective

Supplementary Materials

Table S1. Farms, date and location of sample collection and distribution of the *S. aureus* isolates among cows, farmer and environment samples.

Farm	Date	Location	Sample	Sex	Year of birth	Breed	Isolate
	25.02.2019	Vila Marim	Cow	F	2005	Maronesa	_
	25.02.2019	Vila Marim	Cow	F	2007	Maronesa	_
1	25.02.2019	Vila Marim	Water	_	_	_	_
	25.02.2019	Vila Marim	Soil	_	_	_	_
	25.02.2019	Vila Marim	Farmer	_	_	_	_
	25.02.2019	Vila Marim	Cow	F	2012	Crossbreed	_
	25.02.2019	Vila Marim	Cow	F	2014	Crossbreed	_
	25.02.2019	Vila Marim	Cow	F	2013	Crossbreed	_
	25.02.2019	Vila Marim	Cow	F	2014	Crossbreed	_
	25.02.2019	Vila Marim	Cow	F	2017	Crossbreed	_
2	25.02.2019	Vila Marim	Cow	F	2015	Holstein-Friesian	_
	25.02.2019	Vila Marim	Cow	F	2017	Crossbreed	_
	25.02.2019	Vila Marim	Water	_	_	_	_
	25.02.2019	Vila Marim	Soil	_	_	_	_
	25.02.2019	Vila Marim	Cow	_	_	_	_
	25.02.2019	Vila Marim	Cow	_	_	_	_
	25.02.2019	Vila Marim	Cow	F	2015	Holstein-Friesian	VS3218
	25.02.2019	Vila Marim	Cow	M	2018	Crossbreed	_
	25.02.2019	Vila Marim	Cow	M	2018	Crossbreed	_
3	25.02.2019	Vila Marim	Cow	M	2018	Crossbreed	_
	25.02.2019	AGAREZ	Cow	F	2015	Holstein-Friesian	_
	25.02.2019	Vila Marim	Soil	_	_	_	_
	25.02.2019	Vila Marim	Soil	_	_	_	
	25.02.2019	Vila Marim	Cow	F	2015	Maronesa	_
	25.02.2019	Vila Marim	Cow	F	2012	Maronesa	_
4	25.02.2019	Vila Marim	Water	_	_	_	_
	25.02.2019	Vila Marim	Soil	_	_	_	_
	25.02.2019	Vila Marim	Farmer	_	_	_	_
5	25.02.2019	Vila Marim	Cow	F	2011	Holstein-Friesian	_
	25.02.2019	Vila Marim	Water	_	_	_	_
	25.02.2019	Vila Marim	Soil	_	_	_	_
6	25.02.2019	Vila Marim	Cow	F	2013	Maronesa	
	25.02.2019	Vila Marim	H5	_	_	_	VS3219
	25.02.2019	Vila Marim	Cow	F	2009	Maronesa	_
7	25.02.2019	Vila Marim	Cow	F	2006	Maronesa	_
	25.02.2019	Vila Marim	Water	_	_	_	_

	25.02.2019	Vila Marim	Soil	_	_	_	_
	25.02.2019	Vila Marim	Farmer	_	_	_	_
	25.02.2019	Vila Marim	Farmer	_	_	_	_
	25.02.2019	Vila Marim	Cow	F	2009	Maronesa	_
	25.02.2019	Vila Marim	Cow	F	2009	Maronesa	_
	25.02.2019	Vila Marim	Water	_	_	_	_
8	25.02.2019	Vila Marim	Soil	_	_	_	_
	25.02.2019	Vila Marim	Farmer	_	_	_	_
	25.02.2019	Vila Marim	Farmer	_	_	_	_
	25.02.2019	Vila Marim	Farmer	_	_	_	_
9	25.02.2019	Vila Marim	Cow	F	2018	Crossbreed	_
	04.03.2019	Sirarelhos	Cow	F	2004	Maronesa	VS3220
	04.03.2019	Sirarelhos	Cow	F	2008	Maronesa	VS3221
	04.03.2019	Sirarelhos	Cow	F	2006	Maronesa	VS3222
	04.03.2019	Sirarelhos	Farmer	_	_	_	_
	04.03.2019	Sirarelhos	Farmer	_	_	_	_
	04.03.2019	Sirarelhos	Farmer	_	_	_	_
10	04.03.2019	Sirarelhos	Farmer	_	_	_	_
	04.03.2019	Sirarelhos	Water	_	_	_	_
	04.03.2019	Sirarelhos	Cow	F	2004	Maronesa	_
	04.03.2019	Sirarelhos	Cow	F	2007	Maronesa	_
	04.03.2019	Sirarelhos	Cow	F	2012	Maronesa	_
	04.03.2019	Sirarelhos	Cow	F	2012	Maronesa	_
	04.03.2019	Sirarelhos	Soil	_	_	_	_
	04.03.2019	Sirarelhos	Farmer	_	_	_	_
	04.03.2019	Sirarelhos	Cow	F	2011	Maronesa	_
11	04.03.2019	Sirarelhos	Cow	F	2012	Maronesa	_
	04.03.2019	Sirarelhos	Soil	_	_	_	_
	04.03.2019	Galegos da Serra	Cow	F	2011	Maronesa	_
12	04.03.2019	Galegos da Serra	Cow	F	2009	Maronesa	_
	04.03.2019	Galegos da Serra	Water	_	_	_	_
	04.03.2019	Galegos da Serra	Soil	_	_	_	_
	04.03.2019	Sirarelhos	Cow	F	2008	Maronesa	_
	04.03.2019	Sirarelhos	Cow	F	2010	Maronesa	_
	04.03.2019	Sirarelhos	Cow	F	2015	Maronesa	VS3223
	04.03.2019	Sirarelhos	Cow	F	2002	Maronesa	_
13	04.03.2019	Sirarelhos	Cow	F	2012	Maronesa	VS3224
	04.03.2019	Sirarelhos	Cow	F	2008	Maronesa	VS3225
	04.03.2019	Sirarelhos	Cow	F	2004	Maronesa	_
	04.03.2019	Sirarelhos	Cow	F	2001	Maronesa	VS3226
	04.03.2019	Sirarelhos	Cow	F	2010	Maronesa	
	04.03.2019	Sirarelhos	Cow	F	2017	Maronesa	_
	04.03.2019	Sirarelhos	Water	_	_	_	_
	04.03.2019	Sirarelhos	Soil	_	_	_	_
	04.03.2019	Sirarelhos	Farmer	_	_	_	VS3227
	04.03.2019	Sirarelhos	Farmer	_	_	_	VS3228
	04.03.2019	Sirarelhos	Farmer	_	_	_	VS3229
	04.03.2019	Sirarelhos	Farmer	_	_	_	_
	04.03.2019	Sirarelhos	Cow	M	2016	Maronesa	_
	04.03.2019	Sirarelhos	Cow	F	2007	Maronesa	_
	04.03.2019	Sirarelhos	Cow	F	2006	Maronesa	_
14	04.03.2019	Sirarelhos	Cow	F	2016	Maronesa	_
	04.03.2019	Sirarelhos	Cow	F	2007	Maronesa	_
	04.03.2019	Sirarelhos	Cow	F	2017	Maronesa	_
	04.03.2019	Sirarelhos	Soil	_	_	_	VS3230
	04.03.2019	Sirarelhos	Water	_	_	_	_
15	04.03.2019	Sirarelhos	Cow	F	2010	Maronesa	_
	04.03.2019	Sirarelhos	Cow	F	2013	Maronesa	_

	04.03.2019	Sirarelhos	Cow	F	2011	Maronesa	_
	04.03.2019	Sirarelhos	Cow	F	2017	Maronesa	_
	04.03.2019	Sirarelhos	Cow	F	2016	Maronesa	_
	04.03.2019	Sirarelhos	Cow	F	2017	Maronesa	VS2331
	04.03.2019	Sirarelhos	Cow	F	2013	Maronesa	VS2332
	04.03.2019	Sirarelhos	Cow	F	2004	Maronesa	_
	04.03.2019	Sirarelhos	Cow	F	2012	Maronesa	_
	04.03.2019	Sirarelhos	Cow	F	2005	Maronesa	_
	04.03.2019	Sirarelhos	Cow	F	2010	Maronesa	_
	04.03.2019	Sirarelhos	Cow	F	2010	Maronesa	_
	04.03.2019	Sirarelhos	Soil	_	_	_	_
	04.03.2019	Sirarelhos	Water	_	_	_	_
	04.03.2019	Sirarelhos	Cow	F	2013	Maronesa	_
	04.03.2019	Sirarelhos	Cow	F	2013	Maronesa	_
16	04.03.2019	Sirarelhos	Cow	F	2008	Maronesa	
	04.03.2019	Sirarelhos	Cow	F	2010	Maronesa	VS2333
	04.03.2019	Sirarelhos	Cow	F	2010	Maronesa	VS2334
	04.03.2019	Sirarelhos	Cow	F	2013	Maronesa	_
	04.03.2019	Sirarelhos	Cow	F	2015	Maronesa	_
	04.03.2019	Sirarelhos	Cow	F	2017	Maronesa	_
	04.03.2019	Sirarelhos	Cow	F	2017	Maronesa	VS2335
	04.03.2019	Sirarelhos	Cow	F	2018	Maronesa	_
	04.03.2019	Sirarelhos	Water	_	_	_	_
	04.03.2019	Sirarelhos	Soil	_	_	_	_
	04.03.2019	Sirarelhos	Farmer	_	_	_	VS2336
	04.03.2019	Sirarelhos	Farmer	_	_	_	VS2337
	04.03.2019	Sirarelhos	Farmer	_	_	_	VS2338
	04.03.2019	Sirarelhos	Farmer	_	_	_	VS2339
	04.03.2019	Sirarelhos	Farmer	_	_	_	VS2340
	04.03.2019	Sirarelhos	Farmer	_	_	_	_
	04.03.2019	Sirarelhos	Cow	F	2013	Maronesa	VS2341
	04.03.2019	Sirarelhos	Cow	F	2013	Maronesa	_
17	04.03.2019	Sirarelhos	Cow	F	2009	Maronesa	_
	04.03.2019	Sirarelhos	Cow	F	2011	Maronesa	VS3242
	04.03.2019	Sirarelhos	Cow	F	2013	Maronesa	_
	04.03.2019	Sirarelhos	Cow	F	2016	Maronesa	_
	04.03.2019	Sirarelhos	Cow	F	2006	Maronesa	_
	04.03.2019	Sirarelhos	Cow	F	2006	Maronesa	_
	04.03.2019	Sirarelhos	Soil	_	_	_	_
	04.03.2019	Sirarelhos	Farmer	_	_	_	_
	04.03.2019	Sirarelhos	Farmer	_	_	_	_
18	04.03.2019	Sirarelhos	Cow	F	2018	Crossbreed	_
	04.03.2019	Sirarelhos	Cow	M	2018	Holstein-Friesian	_
19	12.03.2019	Galegos da Serra	Cow	F	2003	Maronesa	_
	12.03.2019	Galegos da Serra	Cow	F	2014	Maronesa	_
	12.03.2019	Galegos da Serra	Water	_	_	_	_
	12.03.2019	Galegos da Serra	Soil	_	_	_	_
	12.03.2019	Galegos da Serra	Farmer	_	_	_	_
	12.03.2019	Galegos da Serra	Farmer	_	_	_	_
20	12.03.2019	Galegos da Serra	Cow	F	2005	Maronesa	_
	12.03.2019	Galegos da Serra	Cow	F	2010	Maronesa	_
	12.03.2019	Galegos da Serra	Cow	F	2014	Maronesa	_
	12.03.2019	Galegos da Serra	Cow	F	2014	Maronesa	_
	12.03.2019	Galegos da Serra	Water	_	_	_	_
	12.03.2019	Galegos da Serra	Soil	_	_	_	_
	12.03.2019	Galegos da Serra	Farmer	_	_	_	_
	12.03.2019	Galegos da Serra	Farmer	_	_	_	VS3243

	12.03.2019	Galegos da Serra	Cow	F	2016	Maronesa	_
	12.03.2019	Galegos da Serra	Cow	F	2011	Maronesa	_
21	12.03.2019	Galegos da Serra	Cow	F	2017	Maronesa	_
	12.03.2019	Galegos da Serra	Soil	_	_	_	_
	12.03.2019	Galegos da Serra	Farmer	_	_	_	_
	12.03.2019	Galegos da Serra	Cow	F	2005	Maronesa	_
22	12.03.2019	Galegos da Serra	Cow	F	2013	Maronesa	_
	12.03.2019	Galegos da Serra	Water	_	_	_	_
	12.03.2019	Galegos da Serra	Soil	_	_	_	_
	12.03.2019	Galegos da Serra	Cow	F	2012	Maronesa	_
	12.03.2019	Galegos da Serra	Cow	F	2011	Maronesa	_
23	12.03.2019	Galegos da Serra	Cow	F	2017	Maronesa	_
	12.03.2019	Galegos da Serra	Water	_	_	_	_
	12.03.2019	Galegos da Serra	Soil	_	_	_	_
	12.03.2019	Galegos da Serra	Farmer	_	_	_	_
	12.03.2019	Galegos da Serra	Cow	F	2015	Maronesa	_
	12.03.2019	Galegos da Serra	Cow	F	2010	Maronesa	_
	12.03.2019	Galegos da Serra	Cow	F	2015	Maronesa	_
	12.03.2019	Galegos da Serra	Cow	F	2008	Maronesa	_
24	12.03.2019	Galegos da Serra	Cow	F	2009	Maronesa	_
	12.03.2019	Galegos da Serra	Cow	F	2010	Maronesa	_
	12.03.2019	Galegos da Serra	Soil	_	_	_	_
	12.03.2019	Galegos da Serra	Water	_	_	_	_
	12.03.2019	Galegos da Serra	Farmer	_	_	_	_
	12.03.2019	Galegos da Serra	Cow	F	2014	Maronesa	_
	12.03.2019	Galegos da Serra	Cow	F	2014	Maronesa	_
	12.03.2019	Galegos da Serra	Cow	F	2012	Maronesa	_
	12.03.2019	Galegos da Serra	Cow	F	2003	Maronesa	_
	12.03.2019	Galegos da Serra	Cow	F	2005	Maronesa	_
	12.03.2019	Galegos da Serra	Water	_	_	_	_
25	12.03.2019	Galegos da Serra	Water	_	_	_	_
	12.03.2019	Galegos da Serra	Soil	_	_	_	_
	12.03.2019	Galegos da Serra	Farmer	_	_	_	_
	12.03.2019	Galegos da Serra	Farmer	_	_	_	_
	12.03.2019	Galegos da Serra	Farmer	_	_	_	_
	12.03.2019	Galegos da Serra	Cow	F	2007	Maronesa	VS324
	12.03.2019	Galegos da Serra	Cow	F	2013	Maronesa	_
	12.03.2019	Galegos da Serra	Cow	F	2004	Maronesa	_
	12.03.2019	Galegos da Serra	Cow	F	2013	Maronesa	_
26	12.03.2019	Galegos da Serra	Soil	_	_	_	_
	12.03.2019	Galegos da Serra	Water	_	_	_	_
	12.03.2019	Galegos da Serra	Farmer	_	_	_	_
	12.03.2019	Relva	Cow	F	2011	Maronesa	_
27	12.03.2019	Relva	Water	_	_	_	_
	12.03.2019	Relva	Soil	_	_	_	_
	12.03.2019	Relva	Farmer	_	_	_	_
	15.03.2019	Lordelo	Cow	F	2013	Crossbreed	_
	15.03.2019	Lordelo	Cow	F	2012	Crossbreed	_
28	15.03.2019	Lordelo	Water	_	_	_	_
	15.03.2019	Lordelo	Soil	_	_	_	_
	15.03.2019	Lordelo	Farmer	_	_	_	_
	15.03.2019	Lordelo	Cow	M	2017	Holstein-Friesian	_
	15.03.2019	Lordelo	Soil	_	_	_	_
29	15.03.2019	Lordelo	Water	_	_	_	_
	15.03.2019	Lordelo	Cow	M	2017	Crossbreed	_
	15.03.2019	Lordelo	Soil	_	_	_	_
	15.03.2019	Lordelo	Farmer	_	_	_	_

	15.03.2019	Lordelo	Farmer	_	_	_	_
30	15.03.2019	Lordelo	Cow	F	2013	Crossbreed	_
	15.03.2019	Lordelo	Cow	F	2015	Crossbreed	_
	15.03.2019	Lordelo	Soil	_	_	_	_
	15.03.2019	Lordelo	Farmer	_	_	_	_
31	15.03.2019	Lordelo	Cow	F	2017	Maronesa	_
	15.03.2019	Lordelo	Water	_	_	_	_
	15.03.2019	Lordelo	Soil	_	_	_	_
	15.03.2019	Lordelo	Farmer	_	_	_	_
	15.03.2019	Lordelo	Farmer	_	_	_	_
32	18.03.2019	Lordelo	Cow	F	2012	Maronesa	_
	18.03.2019	Lordelo	Cow	F	2014	Maronesa	_
	18.03.2019	Lordelo	Cow	F	2014	Maronesa	_
	18.03.2019	Lordelo	Soil	_	_	_	_
	18.03.2019	Lordelo	Water	_	_	_	_
33	18.03.2019	Lordelo	Cow	F	2010	Maronesa	_
	18.03.2019	Lordelo	Cow	F	2013	Maronesa	_
	18.03.2019	Lordelo	Soil	_	_	_	_
	18.03.2019	Lordelo	Water	_	_	_	_
	18.03.2019	Lordelo	Farmer	_	_	_	_
34	18.03.2019	Lordelo	Cow	F	2011	Maronesa	_
	18.03.2019	Lordelo	Cow	F	2012	Maronesa	_
	18.03.2019	Lordelo	Water	_	_	_	_
	18.03.2019	Lordelo	Soil	_	_	_	_
35	18.03.2019	Lordelo	Cow	F	2015	Maronesa	_
	18.03.2019	Lordelo	Cow	F	2014	Maronesa	_
	18.03.2019	Lordelo	Cow	F	2016	Maronesa	_
	18.03.2019	Lordelo	Water	_	_	_	_
	18.03.2019	Lordelo	Soil	_	_	_	_
	18.03.2019	Lordelo	Farmer	_	_	_	_
36	18.03.2019	Lordelo	Cow	F	2010	Maronesa	_
	18.03.2019	Lordelo	Cow	F	2011	Maronesa	_
	18.03.2019	Lordelo	Cow	F	2013	Maronesa	_
	18.03.2019	Lordelo	Water	_	_	_	_
	18.03.2019	Lordelo	Soil	_	_	_	_
	18.03.2019	Lordelo	Farmer	_	_	_	_
37	18.03.2019	Lordelo	Cow	F	2009	Maronesa	_
	18.03.2019	Lordelo	Cow	F	2011	Maronesa	_
	18.03.2019	Lordelo	Cow	F	2008	Maronesa	_
	18.03.2019	Lordelo	Cow	F	2011	Maronesa	_
	18.03.2019	Lordelo	Cow	F	2008	Maronesa	_
	18.03.2019	Lordelo	Cow	F	2013	Maronesa	_
	18.03.2019	Lordelo	Cow	F	2009	Maronesa	_
	18.03.2019	Lordelo	Cow	F	2010	Maronesa	_
	18.03.2019	Lordelo	Cow	F	2015	Maronesa	_
	18.03.2019	Lordelo	Cow	F	2012	Maronesa	_
	18.03.2019	Lordelo	Cow	F	2014	Maronesa	_
	18.03.2019	Lordelo	Cow	F	2013	Maronesa	_
	18.03.2019	Lordelo	Cow	F	2007	Maronesa	_
	18.03.2019	Lordelo	Cow	M	2018	Maronesa	_
	18.03.2019	Lordelo	Water	_	_	_	_
	18.03.2019	Lordelo	Soil	_	_	_	_
38	18.03.2019	Lordelo	Cow	F	2011	Maronesa	_
	18.03.2019	Lordelo	Cow	F	2009	Maronesa	_
	18.03.2019	Lordelo	Water	_	_	_	_
	18.03.2019	Lordelo	Soil	_	_	_	_
	18.03.2019	Lordelo	Cow	F	2014	Maronesa	_
	18.03.2019	Lordelo	Cow	F	2012	Maronesa	_

	18.03.2019	Lordelo	Cow	M	2016	Maronesa	_
	18.03.2019	Lordelo	Water	_	_	_	_
	18.03.2019	Lordelo	Soil	_	_	_	_
39	25.03.2019	Sapiões	Cow	F	2016	Maronesa	VS3245
	25.03.2019	Sapiões	Cow	F	2012	Maronesa	_
	25.03.2019	Sapiões	Cow	F	2014	Maronesa	VS3246
	25.03.2019	Sapiões	Cow	F	2007	Maronesa	VS3247
	25.03.2019	Sapiões	Cow	F	2015	Maronesa	VS3248
	25.03.2019	Sapiões	Farmer	_	_	_	_
	25.03.2019	Sapiões	Farmer	_	_	_	_
40	25.03.2019	Sapiões	Cow	F	2014	Maronesa	_
	25.03.2019	Sapiões	Farmer	_	_	_	_
41	25.03.2019	Sapiões	Cow	F	2013	Crossbreed	_
	25.03.2019	Sapiões	Cow	F	2011	Crossbreed	_
	25.03.2019	Sapiões	Soil	_	_	_	_
42	25.03.2019	Sapiões	Cow	F	2006	Maronesa	_
	25.03.2019	Sapiões	Cow	F	2009	Maronesa	_
	25.03.2019	Sapiões	Cow	F	2015	Crossbreed	_
	25.03.2019	Sapiões	Cow	F	2009	Holstein-Friesian	_
	25.03.2019	Sapiões	Cow	F	2014	Maronesa	VS3249
	25.03.2019	Sapiões	Water	_	_	_	_
	25.03.2019	Sapiões	Soil	_	_	_	_
	25.03.2019	Sapiões	Farmer	_	_	_	_
43	25.03.2019	Gulpilhares	Cow	F	2003	Maronesa	_
	25.03.2019	Gulpilhares	Water	_	_	_	_
	25.03.2019	Gulpilhares	Soil	_	_	_	_
	25.03.2019	Gulpilhares	Farmer	_	_	_	_
44	25.03.2019	Gulpilhares	Cow	F	2015	Crossbreed	_
	25.03.2019	Gulpilhares	Cow	F	2013	Crossbreed	_
	25.03.2019	Gulpilhares	Cow	F	2000	Crossbreed	_
	25.03.2019	Gulpilhares	Water	_	_	_	_
	25.03.2019	Gulpilhares	Soil	_	_	_	_
	25.03.2019	Gulpilhares	Farmer	_	_	_	_
45	25.03.2019	Gulpilhares	Cow	F	2013	Maronesa	_
	25.03.2019	Gulpilhares	Cow	F	2010	Maronesa	_
	25.03.2019	Gulpilhares	Soil	_	_	_	_
	25.03.2019	Gulpilhares	Farmer	_	_	_	_
46	25.03.2019	Gulpilhares	Cow	F	2013	Holstein-Friesian	_
	25.03.2019	Gulpilhares	Soil	_	_	_	_
	25.03.2019	Gulpilhares	Farmer	_	_	_	VS3250
47	25.03.2019	Gulpilhares	1056	F	2007	Holstein-Friesian	VS3251
	25.03.2019	Gulpilhares	Soil	_	_	_	_
	25.03.2019	Gulpilhares	Farmer	_	_	_	VS3252
48	25.03.2019	Gulpilhares	Cow	F	2013	Holstein-Friesian	_
	25.03.2019	Quintelas	Cow	F	2011	Crossbreed	_
	25.03.2019	Gulpilhares	Water	_	_	_	_
	25.03.2019	Gulpilhares	Soil	_	_	_	_
	25.03.2019	Gulpilhares	Farmer	_	_	_	VS3253
	25.03.2019	Quintelas	Cow	F	2006	Maronesa	_
	25.03.2019	Quintelas	Cow	F	2007	Maronesa	_
	25.03.2019	Quintelas	Farmer	_	_	_	VS3254
49	26.03.2019	Mondrões	Cow	M	2018	Maronesa	_
	26.03.2019	Mondrões	Cow	F	2010	Maronesa	_
	26.03.2019	Mondrões	Cow	F	2015	Maronesa	VS3255

	26.03.2019	Mondrões	Cow	F	2013	Maronesa	VS3256
	26.03.2019	Mondrões	Cow	F	2015	Maronesa	_
	26.03.2019	Mondrões	Water	_	_	_	_
	26.03.2019	Mondrões	Soil	_	_	_	_
50	26.03.2019	Mondrões	Cow	F	2010	Maronesa	_
	26.03.2019	Mondrões	Farmer	_	_	_	_
51	26.03.2019	Arrabães	Cow	F	2001	Maronesa	_
	26.03.2019	Arrabães	Cow	F	2002	Maronesa	_
	26.03.2019	Arrabães	Water	_	_	_	_
	26.03.2019	Arrabães	Soil	_	_	_	_
52	01.04.2019	Relva	Cow	F	2007	Maronesa	_
	01.04.2019	Relva	Cow	F	2010	Crossbreed	VS3257
	01.04.2019	Relva	Cow	F	2013	Maronesa	_
	01.04.2019	Relva	Cow	F	2015	Maronesa	_
	01.04.2019	Relva	Cow	F	2015	Crossbreed	_
	01.04.2019	Relva	Cow	F	2016	Crossbreed	_
	01.04.2019	Relva	Cow	F	2013	Crossbreed	VS3258
	01.04.2019	Relva	Cow	F	2015	Crossbreed	_
	01.04.2019	Relva	Cow	F	2008	Maronesa	_
	01.04.2019	Relva	Cow	F	2012	Maronesa	_
	01.04.2019	Relva	Cow	F	2013	Maronesa	_
	01.04.2019	Relva	Cow	F	2017	Maronesa	_
	01.04.2019	Relva	Farmer	_	_	_	VS3259
	01.04.2019	Relva	Farmer	_	_	_	_
	01.04.2019	Relva	Farmer	_	_	_	_
	01.04.2019	Relva	Farmer	_	_	_	VS3260
	01.04.2019	Relva	Farmer	_	_	_	VS3261
	01.04.2019	Relva	Water	_	_	_	_
	01.04.2019	Relva	Soil	_	_	_	_
53	01.04.2019	Borbela	Cow	F	2009	Crossbreed	_
	01.04.2019	Borbela	Farmer	_	_	_	_
	01.04.2019	Borbela	Water	_	_	_	_
	01.04.2019	Borbela	Soil	_	_	_	_
54	01.04.2019	Borbela	Cow	F	2004	Maronesa	_
	01.04.2019	Borbela	Cow	F	2011	Maronesa	_
	01.04.2019	Borbela	Cow	F	2018	Maronesa	_
	01.04.2019	Borbela	Cow	F	2012	Maronesa	_
55	01.04.2019	Borbela	Cow	F	2010	Crossbreed	_
	01.04.2019	Borbela	Cow	F	2003	Crossbreed	_
	01.04.2019	Borbela	Cow	F	2007	Crossbreed	_
	01.04.2019	Borbela	Cow	F	2016	Crossbreed	_
	01.04.2019	Borbela	Water	_	_	_	VS3262
	01.04.2019	Borbela	Soil	_	_	_	_
56	01.04.2019	Borbela	Cow	F	2013	Maronesa	_
	01.04.2019	Borbela	Water	_	_	_	_
	01.04.2019	Borbela	Soil	_	_	_	_
	01.04.2019	Borbela	Farmer	_	_	_	VS3263
57	02.04.2019	Agarez	Cow	F	2015	Maronesa	_
	02.04.2019	Agarez	Cow	F	2005	Maronesa	_
	02.04.2019	Agarez	Cow	F	2017	Maronesa	_
	02.04.2019	Agarez	Cow	F	2013	Maronesa	_
	02.04.2019	Agarez	Cow	F	2010	Maronesa	_
	02.04.2019	Agarez	Cow	F	2007	Maronesa	_
	02.04.2019	Agarez	Cow	M	2018	Maronesa	_
	02.04.2019	Agarez	Cow	F	2018	Maronesa	_
	02.04.2019	Agarez	Water	_	_	_	_
	02.04.2019	Agarez	Soil	_	_	_	_
	02.04.2019	Agarez	Farmer	_	_	_	_

58	02.04.2019	Agarez	Cow	F	2014	Maronesa	_
	02.04.2019	Agarez	Cow	F	2010	Maronesa	_
	02.04.2019	Agarez	Cow	F	2015	Maronesa	_
	02.04.2019	Agarez	Cow	F	2014	Maronesa	_
	02.04.2019	Agarez	Cow	F	2015	Maronesa	_
	02.04.2019	Agarez	Water	_	_	_	_
	02.04.2019	Agarez	Soil	_	_	_	_
	02.04.2019	Agarez	Farmer	_	_	_	VS3264
59	02.04.2019	Agarez	Cow	F	2017	Maronesa	_
	02.04.2019	Agarez	Cow	F	2018	Crossbreed	_
	02.04.2019	Agarez	Cow	F	2018	Crossbreed	_
	02.04.2019	Agarez	Water	_	_	_	_
	02.04.2019	Agarez	Soil	_	_	_	_
	02.04.2019	Agarez	Farmer	_	_	_	_
60	02.04.2019	Agarez	Cow	M	2018	Crossbreed	VS3265
	02.04.2019	Agarez	Cow	M	2018	Crossbreed	VS3266 and VS3267
	02.04.2019	Agarez	Cow	F	2018	Holstein-Friesian	_
	02.04.2019	Agarez	Water	_	_	_	_
	02.04.2019	Agarez	Soil	_	_	_	_
61	02.04.2019	Agarez	Cow	F	2015	Maronesa	_
	02.04.2019	Agarez	Water	_	_	_	_
	02.04.2019	Agarez	Soil	_	_	_	_
62	02.04.2019	Agarez	Cow	F	2015	Maronesa	_
	02.04.2019	Agarez	Cow	F	2016	Maronesa	_
	02.04.2019	Agarez	Cow	F	2010	Maronesa	VS3268
	02.04.2019	Agarez	Cow	F	2011	Maronesa	VS3269
	02.04.2019	Agarez	Water	_	_	_	_
	02.04.2019	Agarez	Soil	_	_	_	_
	02.04.2019	Agarez	Farmer	_	_	_	VS3270
	02.04.2019	Agarez	Farmer	_	_	_	VS3271
63	02.04.2019	Agarez	Cow	M	2017	Maronesa	VS3272
	02.04.2019	Agarez	Cow	M	2017	Maronesa	_
	02.04.2019	Agarez	Cow	F	2017	Maronesa	_
	02.04.2019	Agarez	Cow	F	2017	Maronesa	_
	02.04.2019	Agarez	Cow	F	2011	Maronesa	_
	02.04.2019	Agarez	Cow	F	2004	Maronesa	VS3273
	02.04.2019	Agarez	Cow	F	2008	Maronesa	_
	02.04.2019	Agarez	Cow	F	2016	Maronesa	_
	02.04.2019	Agarez	Cow	F	2014	Maronesa	_
	02.04.2019	Agarez	Cow	F	2016	Maronesa	_
	02.04.2019	Agarez	Cow	F	2012	Maronesa	_
	02.04.2019	Agarez	Cow	F	2009	Maronesa	_
	02.04.2019	Agarez	Cow	F	2016	Maronesa	_
	02.04.2019	Agarez	Water	_	_	_	_
	02.04.2019	Agarez	Soil	_	_	_	_
	02.04.2019	Agarez	Farmer	_	_	_	_
	02.04.2019	Agarez	Farmer	_	_	_	_
64	02.04.2019	Agarez	Cow	F	2015	Crossbreed	VS3274
	02.04.2019	Agarez	Water	_	_	_	_
	02.04.2019	Agarez	Soil	_	_	_	_
	02.04.2019	Agarez	Farmer	_	_	_	VS3275

Table S2. Primer pairs used for molecular typing and detection of antimicrobial resistance genes in *S. aureus* strains.

Gene (Amplicon Size)	Sequence (5′ › 3′)	Conditions	Reference
*mec*A (527 bp)	F: GGGATCATAGCGTCATTATTC<br>R: AACGATTGTGACACGATAGCC	94 ºC 5 min 1 cycle<br>94 ºC 30 sec<br>55 ºC 30 sec 30 cycles<br>72 ºC 1 min<br>72 ºC 10 min 1 cycle	[77]
*bla*Z (772 bp)	F: CAGTTCACATGCCAAAGAG<br>R: TACACTCTTGGCGGTTTC	94 ºC 3 min 1 cycle<br>94 ºC 1 min<br>50 ºC 1 min 30 cycles<br>72 ºC 2 min<br>72 ºC 5 min 1 cycle	[78]
*erm*A (645 bp)	F: TCTAAAAAGCATGTAAAAGAA<br>R: CTTCGATAGTTTATTAATATTAG	93 ºC 3 min 1 cycle<br>93 ºC 1 min<br>52 ºC 1 min 35 cycles<br>72 ºC 1 min<br>72 ºC 5 min 1 cycle	[79]
*erm*B (639 bp)	F: GAAAAGTACTCAACCAAATA<br>R: AGTAACGGTACTTAAATTGTTTA		[37]
*erm*C (642 bp)	F: TCAAAACATAATATAGATAAA<br>R: GCTAATATTGTTTAAATCGTCAAT		[79]
*erm*T (200 bp)	F: CCGCCATTGAAATAGATCCT<br>R: TTCTGTAGCTGTGCTTTCAAAAA	94 ºC 3 min 1 cycle<br>94 ºC 1 min<br>55 ºC 1 min 30 cycles<br>72 ºC 1 min<br>72 ºC 5 min 1 cycle	[80]
msr(A/B) (399 bp)	F: GCAAATGGTGTAGGTAAGACAACT<br>R: ATCATGTGATGTAAACAAAAT	95 ºC 3 min 1 cycle<br>93 ºC 30 sec<br>55 ºC 2 min 35 cycles<br>72 ºC 1,5 min<br>72 ºC 5 min 1 cycle	[81]
mph(C) (900 bp)	F: ATGACTCGACATAATGAAAT<br>R: CTACTCTTTCATACCTAACTC	94 ºC 3 min 1 cycle<br>94 ºC 1 min<br>45 ºC 1 min 30 cycles<br>72 ºC 1 min<br>72 ºC 5 min 1 cycle	[78]
lnu(A) (323 bp)	F: GGTGGCTGGGGGGTAGATGTATTAACTGG<br>R: GCTTCTTTTGAAATACATGGTATTTTTCGATC	94 ºC 2 min 1 cycle<br>94 ºC 30 sec<br>57 ºC 30 sec 30 cycles<br>72 ºC 1 min<br>72 ºC 10 min 1 cycle	[82]
lnu(B) (944 bp)	F: CCTACCTATTGTTTGTGGAA<br>R: ATAACGTTACTCTCCTATTC	94 ºC 5 min 1 cycle<br>94 ºC 45 sec<br>54 ºC 45 sec 30 cycles<br>72 ºC 1 min	[83]

		72 ºC 5 min 1 cycle<br>94 ºC 3 min 1 cycle	
vga(A)<br>(1264 bp)	F: AGTGGTGGTGAAGTAACACG<br>R: GGTTCAATACTCAATCGACTGAG	94 ºC 1 min<br>56 ºC 1 min 30 cycles<br>72 ºC 1 min	[84]
		72 ºC 5 min 1 cycle<br>94 ºC 1 min 1 cycle	
vga(B)<br>(576 pb)	F: TGACAATATGAGTGGTGGTG<br>R: GCGACCATGAAATTGCTCTC	94 ºC 1 min<br>55 ºC 2 min 30 cycles<br>72 ºC 2 min	[85]
*tet*K<br>(697 bp)	F: TTAGGTGAAGGGTTAGGTCC<br>R: GCAAACTCATTCCAGAAGCA	72 ºC 10 min 1 cycle<br>94 ºC 3 min 1 cycle	[86]
*tet*M<br>(576 bp)	F: GTTAAATAGTGTTCTTGGAG<br>R: CTAAGATATGGCTCTAACAA	94 ºC 1 min<br>54 ºC 1 min 30 cycles<br>72 ºC 1 min	[86]
*tet*O<br>(615 bp)	F: GATGGCATACAGGCACAGAC<br>R: CAATATCACCAGAGCAGGCT	72 ºC 5 min 1 cycle<br>94 ºC 1 min 1 cycle	[86]
*tet*L<br>(456 bp)	F: CATTTGGTCTTATTGGATCG<br>R: ATTACACTTCCGATTTCGG	94 ºC 1 min<br>50 ºC 1 min 30 cycles<br>72 ºC 1 min	[86]
		72 ºC 10 min 1 cycle<br>94 ºC 5 min 1 cycle	
aac(6′)-Ie-<br>*aph*(2′′)-Ia<br>(220 bp)	F: CCAAGAGCAATAAGGGCATA<br>R: CACTATCATAACCACTACCG	94 ºC 30 sec<br>60 ºC 45 sec 30 cycles<br>72 ºC 2 min	[87]
		72 ºC 7 min 1 cycle<br>94 ºC 3 min 1 cycle	
ant(4′)-Ia<br>(160 bp)	F: GCAAGGACCGACAACATTTC<br>R: TGGCACAGATGGTCATAACC	94 ºC 30 sec<br>60 ºC 45 sec 30 cycles<br>72 ºC 2 min	[87]
		72 ºC 5 min 1 cycle<br>94 ºC 3 min 1 cycle	
str<br>(646 bp)	F: TATTGCTCTCGAGGGTTC<br>R: CTTTCTATATCCATTCATCTC	94 ºC 1 min<br>50 ºC 1 min 30 cycles<br>72 ºC 1 min	[78]
		72 ºC 5 min 1 cycle<br>94 ºC 3 min 1 cycle	
aph(3′)-III<br>(292 bp)	F: GCCGATGTGGATTGCGAAAA<br>R: GCTTGATCCCCAGTAAGTCA	94 ºC 30 sec<br>60 ºC 45 sec 30 cycles<br>72 ºC 2 min	[87]
		72 ºC 5 min 1 cycle	

		94 ºC 1 min 1 cycle	
*fex*A (1272 bp)	F: GTACTTGTAGGTGCAATTACGGCTGA<br>R: CGCATCTGAGTAGGACATAGCGTC	94 ºC 1 min<br>48 ºC 2 min 34 cycles<br>72 ºC 3 min	[88]
		72 ºC 7 min 1 cycle<br>94 ºC 7min<br>min 1 cycle	
*fex*B (816 bp)	F: TTCCCACTATTGGTGAAAGGAT<br>R: GCAATTCCCTTTTATGGACGTT	94 ºC 1 min<br>55 ºC 1 min 30 cycles<br>72 ºC 1 min	[89]
		72 ºC 10 min 1 cycle	
cat$_{pC194}$ (570 bp)	F: CGACTTTTAGTATAACCACAGA<br>R: GCCAGTCATTAGGCCTAT	94 ºC 3 min 1 cycle	[78]
cat$_{pC221}$ (434 bp)	F: ATTTATGCAATTATGGAAGTTG<br>R: TGAAGCATGGTAACCATCAC	94 ºC 1 min<br>50 ºC 1 min 30 cycles<br>72 ºC 1 min	[78]
cat$_{pC223}$ (283 bp)	F: GAATCAAATGCTAGTTTTAACTC<br>R: ACATGGTAACCATCACATAC		[78]
		72 ºC 5 min 1 cycle<br>94 ºC 3 min 1 cycle	
cfr (746 bp)	F: TGAAGTATAAAGCAGGTTGGGAGTCA<br>R: ACCATATAATTGACCACAAGCAGC	94 ºC 1 min<br>56 ºC 1 min 30 cycles<br>72 ºC 1 min	[88]
		72 ºC 5 min 1 cycle<br>94 ºC 3 min 1 cycle	
*fus*B (431 bp)	F: CTATAATGATATTAATGAGATTTTTGG<br>R: TTTTTACATATTGACCATCCGAATTGG		
		94 ºC 30 sec<br>57 ºC 30 sec 25 cycles<br>72 ºC 45 sec	[90]
*fus*C (332 bp)	F: TTAAAGAAAAAGATATTGATATCTCGG<br>R: TTTACAGAATCCTTTTACTTTATTTGG		
		72 ºC 7 min 1 cycle<br>94 ºC 7 min 1 cycle	
*fus*D (456 bp)	F: AATTCGGTCAACGATCCC<br>R: GCCATCATTGCCAGTACG	94 ºC 30 sec<br>57 ºC 30 sec 30 cycles<br>72 ºC 30 sec	[91]
		72 ºC 10 min 1 cycle	
*dfr*A (374 bp)	F: CCTTGGCACTTACCAAATG<br>R: CTGAAGATTCGACTTCCC	94 ºC 3 min 1 cycle	
		94 ºC 1 min<br>50 ºC 1 min 30 cycles<br>72 ºC 1 min	[78]
*dfr*D (582 bp)	F: TTCTTTAATTGTTGCGATGG<br>R: TTAACGAATTCTCTCATATATATG		
		72 ºC 5 min 1 cycle<br>94 ºC 3 min 1 cycle	
*dfr*G (323 bp)	F: TCGGAAGAGCCTTACCTGACAGAA<br>R: CCCTTTTTGGGCAAATACCTCATTCCA	94 ºC 1 min<br>58 ºC 1 min 30 cycles<br>72 ºC 1 min	[80]
		72 ºC 5 min 1 cycle	
*dfr*K	F: GAGAATCCCAGAGGATTGGG	94 ºC 3 min 1 cycle	

(423 bp)	R: CAAGAAGCTTTTCGCTCATAAA	
		94 ºC 1 min
		56 ºC 1 min 30 cycles
		72 ºC 1 min
		72 ºC 5 min 1 cycle
